# Invitations and incentives: a qualitative study of behavioral nudges for primary care screenings in Armenia

**DOI:** 10.1186/s12913-020-05967-z

**Published:** 2020-12-01

**Authors:** Estelle Gong, Adanna Chukwuma, Emma Ghazaryan, Damien de Walque

**Affiliations:** 1 Mount Sinai Health System, New York, NY 10019 USA; 2 World Bank Group, Washington, DC 20433 USA

**Keywords:** Screening, Primary care, Financial incentives, Behavioral economics, Cash transfers, Descriptive social norm, Nudge, Hypertension, Diabetes

## Abstract

**Background:**

Non-communicable diseases account for a growing proportion of deaths in Armenia, which require early detection to achieve disease control and prevent complications. To increase rates of screening, demand-side interventions of personalized invitations, descriptive social norms, labeled cash transfers, and conditional cash transfers were tested in a field experiment. Our complementary qualitative study explores factors leading to the decision to attend screening and following through with that decision, and experiences with different intervention components.

**Methods:**

Informed by the Health Belief Model as our conceptual framework, we collected eighty in-depth interviews with service users and twenty service providers and analyzed them using open coding and thematic analysis.

**Results:**

An individual’s decision to screen depends on 1) the perceived need for screening based on how they value their own health and perceive hypertension and diabetes as a harmful but manageable condition, and 2) the perceived utility of a facility-based screening, and whether screening will provide useful information on disease status or care management and is socially acceptable. Following through with the decision to screen depends on their knowledge of and ability to attend screenings, as well as any external motivators such as an invitation or financial incentive.

**Conclusions:**

Personalized invitations from physicians can prompt individuals to reconsider their need for screening and can, along with financial incentives, motivate individuals to follow through with the decision to screen. The effect of descriptive social norms in invitations should be further studied. Efforts to increase preventive screenings as an entry point into primary care in Armenia may benefit from implementation of tailored messages and financial incentives.

**Trial registration:**

The protocol was approved on January 11, 2019 by the Institutional Review Board of the Center of Medical Genetics and Primary Health Care in Armenia (02570094). https://www.socialscienceregistry.org/trials/3776.

## Background

Globally, over 15 million people die prematurely every year due to non-communicable diseases (NCDs) and around 85% of those deaths occur in low- and middle-income countries (LMICs) [1, 2]. The rise of NCDs, which includes cardiovascular diseases, cancers, respiratory diseases, and diabetes, is due to multiple socio-economic factors that include aging, exposure to tobacco, and unhealthy lifestyles. At the individual level, these socio-economic factors can impact metabolic risk factors like high blood pressure and high blood glucose, which may be diagnosed and managed as hypertension and diabetes mellitus, respectively [2]. Diabetes mellitus, in addition to its own health consequences such as nerve damage and diabetic retinopathy if improperly managed, joins hypertension in increasing the risk of death from cardiovascular diseases such as heart attack and stroke [3–5].

Detecting hypertension and diabetes mellitus is essential to initiating treatment, achieving disease control, and delaying the onset of other NCDs. However, a high proportion of people living with hypertension and diabetes mellitus have never been diagnosed, particularly in LMICs. Of the 1.4 billion people living with diabetes globally, only half are aware of their diagnosis and more than 80% of undiagnosed cases live in LMICs [6]. Only 39.2% of people living with hypertension in LMICs have ever been diagnosed, contributing to the low proportion (10.3%) that achieve control of their blood pressure [7]. Screening through medical examinations and tests increases opportunities to diagnose hypertension and diabetes, even when individuals are asymptomatic or have mild symptoms. Furthermore, screening of high-risk populations can be cost-effectively delivered in primary care facilities or by community health workers even in LMICs [8, 9].

In Armenia, a middle-income country in the South Caucasus, NCDs account for 93% of deaths in the country, including cardiovascular diseases (55.2%), cancers (20.2. percent), and diabetes mellitus (1.5%) [10]. In this small country with a population of 2.9 million, premature mortality rates from diabetes mellitus (560.4 per 100,000 population) are higher than the average in countries with similar sociodemographic indicators (222.2 per 100,000 population), including Azerbaijan (465.6 per 100,000 population) and Georgia (429.9 per 100,000 population) within the South Caucasus region [11]. The high burden of NCDs results in part from gaps in access to and use of high-quality health care, preventing the promotion of healthy lifestyles, early diagnosis, and prevention of complications.

General practitioners and family physicians at the primary care level are responsible for health promotion, screening, treatment, monitoring, and referral to specialist care in Armenia. Primary care services are provided for free at polyclinics in urban areas, ambulatory facilities in rural areas, and outpatient health centers. However, the costs of outpatient medicines and expensive diagnostic care are not covered except for specific groups such as military personnel and their families or those classified as socially vulnerable, such as veterans, disabled individuals, and children of a certain age or disadvantaged households [12–14]. Hence, one in five Armenians still report that financial barriers are reasons for forgoing care. As a result, Armenians average 4 annual outpatient visits per person, below the European average of 7.1 visits [15]. Primary care use may be deterred by the perception that primary care is of poor quality and lacks regulation [16].

To increase utilization of primary care, the Armenian government collaborated with the World Bank and other donors to launch, in 2013, supply-side reforms such as performance-based financing and clinical guideline development and a demand-side mass media campaign educating the public on the benefits of attending screenings. However, despite these ongoing programs, screening rates were persistently low. In 2016, only 43.5% of Armenians over 15 years old had their blood pressure measured by a health provider and only 24% had their blood glucose levels measured in the past 12 months [16]. Focus group discussions with service users and providers highlighted potential barriers to screening, including the underestimation of the harmful effects of hypertension and diabetes and the high perceived costs of attending screenings and post-diagnosis care [17].

The empirical literature highlights the potential for behavioral “nudges” such as physician reminders, invitation letters, and financial incentives to increase preventive health care use. For example, studies show that invitations to attend health checks or screenings for specific diseases such as cancer can increase attendance compared to no intervention [18–20]. The contents of invitations have been investigated as well, from inclusion of a provider signature to descriptive social norms that aim to influence individual behaviors by referencing peer behaviors [18, 21]. Descriptive social norms messaging has been shown to influence behaviors in other settings such as charitable giving [22], voting [23], and water and energy consumption [24]. In health behavior applications, descriptive social norms have been used to encourage healthy lifestyle habits [25–28] and increased uptake of health services like health checks and screenings [21, 29]. Financial incentives such as cash transfers and vouchers have also been shown to increase preventive health care use and even health outcomes in settings of low- and middle-income countries [30–33]. However, there is mixed evidence on the relative impact of conditional and unconditional cash transfers as well as their acceptability among target populations [34–37].

Given the evidence on messages and incentives to increase preventive care use, the low screening rates in Armenia, and the focus group discussion findings highlighting low perceived risk of hypertension and diabetes and high perceived costs, there is a need to assess whether such interventions may be effective in increasing screening attendance in Armenia. Currently, there is a gap in the literature on the impact of behavioral interventions on primary health care use in the Armenian context. In addition to the potential quantitative impact on screening rates, there is an opportunity for qualitative investigation to understand user and provider experiences with such interventions and determine the mechanism of influence that these interventions have on health behaviors in Armenia.

This qualitative study complemented a randomized control trial that was conducted in four provinces in Armenia to evaluate the impact of financial and non-financial demand-side personalized incentives to increase screening attendance for hypertension and diabetes at primary care centers in Armenia [38]. Two thousand individuals aged 35 to 68 who had not screened in the previous year were randomly selected from Armenia’s national e-health database and randomized to one of the four intervention groups or the control group. All intervention groups were delivered a verbal message and written letter that varied by group. The verbal message was delivered based on a script in a written guide, which all fieldworkers were trained to use for consistency in each intervention group. The intervention delivery was verified as part of the process evaluation implemented in parallel with this qualitative study. We describe the content of tested interventions below, while full invitation templates are available in Additional File 1.

Intervention group 1 received a personal invitation that was delivered in person by study fieldworkers, which consisted of a verbal message and printed letters signed by a physician. The letter invited individuals to screen for hypertension and diabetes, highlighted the importance of screening for one’s health, noted that screening was free of charge and not time consuming, and provided the address and phone of the community medical facility where an appointment could be made.

Intervention group 2 received the same invitation as group 1, but the verbal invitation and letter included additional information on the number of men and women in the individual’s peer group that had screened in their communities. Peer groups were defined within three age bands: 35–45 years, 46–55 years, and 56–68 years.

Intervention group 3 received the same invitation as group 1 and a uniquely numbered, single use pharmacy voucher and instructions for use. The letter identified the two pharmacy chains where the voucher could be redeemed for themselves or another person, the choice of products, and the monetary value of AMD 5000 (USD 10). The offer of the voucher was labeled as an encouragement to screen for hypertension and diabetes.

Intervention group 4 received the same invitation as group 1 and instructions that screening could be reported to a designated research assistant, who, upon verifying attendance with the local facility, would provide them a pharmacy voucher. The voucher was identical to the one provided to group 3, except that it was not labeled as an encouragement to screen, but rather as a conditional voucher.

The control group did not receive any intervention under this study, including information and pharmacy vouchers. However, the ongoing mass media campaign funded through the Ministry of Health provided information on the prevalence of diabetes and hypertension and encouraged adults to screen for free for hypertension and diabetes in the local primary care clinic. These campaigns involved broadcasts on public and private television stations, billboards on major roads, posters in health facilities and post offices, and text messages from the Ministry of Health.

The first three interventions increased the probability of screening for both hypertension and diabetes by about 15 percentage points compared to the control group, while the conditional vouchers in intervention group 4 doubled the size of that impact. The conditional vouchers were equally cost-effective as messaging interventions, while the unconditional vouchers were less cost-effective than the other interventions [39]. To inform an understanding of the mechanisms underlying screening behavior in the field experiment, we undertook a qualitative study. Hence, the objective of the study is to understand the factors influencing the decision to attend screenings and following up with that decision, and experiences with the intervention components.

## Methods

### Conceptual framework

This study’s conceptual framework was informed by the Health Belief Model (HBM), which originated in preventive health programs led by the United States Public Health Service in the 1950s and 1960s [40]. In this model, an individual’s likelihood to take action to avoid disease depends on five dimensions: *perceived susceptibility* to the disease, *perceived severity* of the disease, *perceived benefit* of an action in reducing severity or susceptibility, *perceived barriers* to taking action, and the *cue to action*. The *cue to action* is a factor that leads an individual to actually change their behavior, which may or may not be consistent with their perceptions of susceptibility or severity. The cue to action can be internal, such as the experience of a symptom, or external, such as a media campaign or encouragement from a friend. In the 1980s, the dimension of *self-efficacy* was added, as applications of the HBM expanded from one-time preventative actions (such as vaccinations) to more long-term behaviors to prevent and manage chronic disease [41]. Individuals require a greater sense of self-efficacy, or sense of competency, to carry through more complex behavior changes like smoking cessation or medication adherence.

Because the HBM provides the conceptual language to explain not only if a person can and wants to take action, but also if they *will* take action, it is an appropriate framework for our study. In our review of other health behavior theories and models, there was significant overlap with the HBM [42]^.^ For example, the Theory of Planned Behavior can be useful in exploring an individual’s desire and intention to act on a specific health behavior, where a certain behavior depends on an intention that is informed by individual attitudes, subjective norms, and perceived behavioral control [43]. Compared to the HBM, individual attitudes and subjective norms contribute to perceptions of susceptibility, severity, and benefits of action, and perceived behavioral control overlaps with an individual’s sense of self-efficacy.

Using the HBM, we assume that an individual is more likely to attend screening if they perceive themselves as susceptible to hypertension or diabetes, perceive hypertension or diabetes as sufficiently severe, and perceive screening attendance to provide benefits in terms of prevention or management of disease. Whether they attend also depends on individual factors such as their sense of self efficacy in traveling to the clinic and potentially initiating care, and whatever may be their cue to action. Hence, the intervention arms were hypothesized to influence screening behavior through mechanisms delineated in the HBM.

A personalized invitation may serve as a cue to action and increase a sense of self efficacy. It may also increase the perceived susceptibility to and severity of hypertension and diabetes, and subsequently the perceived benefits of screening. The invitation may reduce perceived access barriers by clarifying where and how to attend screening. Learning of peer screening attendance rates may influence screening behavior by demonstrating differences in peer perceptions of severity, susceptibility, and benefits of screening. It may serve as a cue to action and reduce perceived barriers related to social norms around seeking care, which can also add to a sense of self efficacy. A pharmacy voucher may serve as a cue to action, with a stronger effect for vouchers conditional on screening attendance. Vouchers may also address any perceived barriers related to costs of attending screening and add to a sense of self-efficacy (by reducing the burden of medication costs) or signal the value (and benefit) of screening that impacts the perceived severity of or susceptibility to hypertension and diabetes.

### Study design

Our study uses thematic analysis to understand the factors impacting the decision to attend screenings and following up with that decision, and experiences with different intervention arms. Our conceptual framework informed our approach to data collection and analysis, namely in the interview guide design and coding process.

### Data collection

We conducted a total of 100 one-on-one in-depth interviews: 80 with service users who participated in the trial and 20 with service providers at facilities providing screening services for participants in the trial. Service provider perspectives on screening behaviors may differ from those of service users and were included to triangulate findings and identify areas of convergence or divergence. Service users from all intervention arms were equally represented. However, control group service users were not included because the qualitative study was conducted before the endline quantitative survey for the main study. This was done to avoid contamination of experimental groups with qualitative interviews that provided information about other groups.

Interviews were conducted with guides that were developed and reviewed by the study team with expertise in behavioral economics, medicine, health services research, sociology, impact evaluation, social work, and qualitative research. Interview guide content was informed by concepts in the HBM and were pre-tested with purposively selected individuals that were representative of the target population. Wording and cues were revised based on testing feedback. The guides are available in Additional File 2.

Interviews were conducted in January to March 2020 by a team of five Armenian facilitators who were selected based on education (higher education degree in a social science field) and their experience with qualitative interviewing (minimum of 3 years). Facilitators did not have prior contact with study facilities or target communities of the screening intervention. Facilitators also had no knowledge of the quantitative outcomes of the trial and endline screening rates for each intervention group. Facilitators were assigned to interview service users of the same gender to encourage open discussion during interviews.

Facilitators contacted selected participants by phone and scheduled an interview. Even though the study service users were already involved in the trial, the facilitators explained the purpose of their follow up visit using an introductory script. At the time of interviewing, verbal consent was obtained for permission to audio record and transcribe the interview. For service users, interviews took place at home, at work, or in some cases, in the facilitator’s vehicle based on participant preference. Despite attempts to ensure privacy for interviews taking place in the home, at times this was impossible due to space limitations and multiple occupants of the household. Whether others were present for the interview, as well as the general demeanor of the participant, was noted by facilitators. Provider interviews were conducted at facilities, typically in provider offices or another office setting.

Interviews were transcribed verbatim in Armenian, and then summarized in English with supporting quotes. Transcription quality checks were conducted by random validation of transcripts against audio recordings. All selected respondents were interviewed despite achieving data saturation before all the interviews were completed.

### Data analysis

The analytical team was comprised of a health policy specialist with a medical degree and doctoral training in health systems, an Armenian physician with a Master of Public Health, an economist, and a health services researcher with a Master of Science in Global Health. Two members of the analytical team independently conducted open coding of all interview summaries, respectively using Dedoose [44] and manual coding. This initial open coding was done separately to avoid bias in code development. Codes were created by assigning phrases capturing the main concept of the statement that related to central research question: what factors influence the decision to screen and following up on that decision? [45] Following review by the study team of both independent code trees, the code tree was revised to reconcile differences and grouped codes into themes reflecting overarching ideas that explained screening behavior of service users. The team then revised the themes and resolved any disagreements, presenting the results below in accordance with the consolidated criteria for reporting qualitative research (COREQ) [46].

## Results

Table 1 shows characteristics of the sample of service users and service providers. Interviews were conducted with 80 service users, who were evenly distributed across gender and screening status. Ages ranged from 35 to 69, with about half under the age of 50. Of the 20 interviewed service providers, 55% (*n* = 11) were above the age of 60, 80% were female (*n* = 16) and 45% (*n* = 9) held the title of ambulatory or polyclinic director. Average duration of interviews was 25 min (range of 10 to 41 min). No interviews were partially conducted or repeated.

**Table 1:** Sample Characteristics

	Service Users (*n* = 80)		Service Providers (*n* = 20)
Variable	Screened (n = 40)	Not Screened (*n* = 40)	
Gender, n (%)			
Male	20 (25)	20 (25)	4 (20)
Female	20 (25)	20 (25)	16 (80)
Age, n (%)			
30–39	8 (10)	8 (10)	
40–49	12 (15)	12 (15)	6 (30)
50–59	11 (13.75)	11 (13.75)	3 (15)
60–69	9 (11.25)	9 (11.25)	11 (55)
Marz, n (%)			
Ararat	6 (7.5)	7 (8.8)	4 (20)
Armavir	8 (10)	9 (11.3)	5 (25)
Kotayk	11 (13.8)	7 (8.8)	6 (30)
Lori	15 (18.8)	17 (21.3)	5 (25)
Years in Service, n (%)			
10–19			4 (20)
20–29			3 (15)
30–39			8 (40)
40–49			5 (25)
Position, n (%)			
Facility Director			9 (45)
Family Physician, General Practitioner or Therapist			11 (55)

There were 26 service users who were contacted for interviewing and declined to participate. The reasons for refusal to participate were being busy (*n* = 12), being out of the community at the time of contact (*n* = 8), having no desire to participate (*n* = 4), or being ill (*n* = 2).

We identified three major themes that appear to explain the decision to screen (Table 2). First, an individual’s decision to screen depends on the perceived need for screening based on how they value their own health and perceive hypertension and diabetes as a harmful but manageable condition. In addition, an individual’s decision to screen depends on the perceived utility of a facility-based screening, and whether screening will provide useful information on disease status or care management and is socially acceptable. Finally, if an individual perceives they need to be screened, and perceives the facility-based screening as useful, their follow-through depends on their knowledge of and ability to attend screenings, as well as any external motivators such as an invitation or financial incentive.

**Table 2:** Themes and sub-themes

Themes	Sub-themes
Theme 1: The decision to attend screening is more likely with greater perceived need for screening	Prioritizing one’s health
	Underestimating the harmful consequences of hypertension and diabetes
	Believing hypertension can and should be prevented or managed
	Feeling cared for and being reminded of preventative screening benefits after receiving invitations
Theme 2: The decision to attend screening is more likely with greater perceived utility of and access to screenings.	Seeking information on disease status and management
	Trusting the health system
	Being reminded of information to be gained in screening and increasing trust in the health system after receiving invitations
Theme 3: External motivators increase the likelihood of following through with the decision to screen	Being reminded to attend screenings despite time costs after receiving invitations
	In groups 3 and 4, being motivated to screen due to receiving vouchers as a form of assistance

### Theme 1: the decision to attend screening is more likely with greater perceived need for screening

Service users described their decision to screen based on whether they perceive it was needed or not. This need appears to reflect how individuals value their own health, if they perceive hypertension and diabetes as a threat to their health, and if they believe that hypertension and diabetes are conditions to be prevented or managed.

Priority to one’s health and concerns for any deviations from full health appeared to be a motivation for undertaking screenings. About a third (30/100) of respondents reflected on attitudes towards one’s health, with seventeen describing indifference towards one’s well-being as a reason to not seek care. Over half (55/100) of respondents described delaying care until symptoms are extreme, with eighteen attributing the delay to an Armenian mindset or as six of those described, “waiting until the knife hits the bone.” Some reasoned that this indifference results from the assumption that any symptoms or discomfort will pass with time. A third of service users (28/80) expressed that family influence could help overcome this indifference and encourage care seeking, while a similar number (26/80) disagreed and were adamant that “no one” could change their behavior—only they can decide for themselves. When asked about whether gender impacted health seeking, 45 service users thought women may attend more and provided reasons such as women caring more about their health, having more health concerns, or having more time.

*In general, I am very indifferent to myself. Even when I want to visit a doctor, there is always an obstacle. I don’t care much of myself. Sometimes I want to go, but then I say: ‘Screw this, let others go’. I don’t know. (Female, not screened, Group 1)*

*Whoever views health another way, they do their best to frequently go [for checks] and find out... Those people are very few, perhaps only about 10% of our society, who actually go. Others – the majority – don’t go and don’t even want to, and attend only in extreme conditions. (Male, screened, Group 4)*

*I’ve always said: ‘God forbids such situations, when you have to visit a doctor.’ We are Armenians, we think like that and don’t take care much for ourselves. They say, ‘God forbids such thing to happen, when the knife has reached the bones and you have to visit a doctor’. Armenians think like that in general. (Female, not screened, Group 1)*

Underestimating the harmful consequences of hypertension and diabetes reduces the perceived need for screening. Over half of service users (51/80) recognize the widespread prevalence of hypertension or diabetes, and either have one of the conditions themselves or know someone who does. However, a similar proportion (50/80) perceive hypertension and diabetes screening as unnecessary if there are no symptoms, with five service providers also describing this sentiment from their patients. This perception suggests that individuals do not fully appreciate the asymptomatic nature of early hypertension or diabetes, where one may feel normal but screen positive upon examination. A third of service users (28/80) also described the preference to monitor and treat any symptoms at home, either with medication from the pharmacy or with home remedies.

*I already know the symptoms of diabetes and hypertension. If I notice one of the symptoms, I will go for screenings. Otherwise, I won’t go. (Female, screened, Group 2)*

*My husband’s blood pressure is high, mine is low, but we measure it anyway. I measure it in case of headache, just want to know if it is high or low. We do it just to know it is high or low and what we can do for it at home at that moment. (Female, screened, Group 4)*

*I have read that you shouldn’t use chemical soaps or medicine when you have diabetes. Then, some people were saying that legs will rest and hurt less if you put them in salty water […] It is written in the book that if you put 2 cloves under your tongue for 15 days, it regulates your blood sugar. But I haven’t tried it yet.” (Male, screened, Group 3)*

Individuals may be more likely to screen if they perceive hypertension and diabetes as conditions that can and should be prevented or managed. About a quarter (24/80) of service users thought that **it is preferable to prevent conditions** such as hypertension and diabetes, rather than manage or “cure” it once diagnosed. Five service providers commented on the need for people to attend screenings for preventative purposes rather than curative purposes, but that this attitude has only recently started to change.

*Of course, the preventive is right. When I heal the plants in my field we start with the preventive, because when the plant is ill, we’ll have big financial losses. The same is for people, just like in nature, if we don’t do preventive healing, productivity greatly decreases. (Male, screened, Group 1)*

*If people know they have disease they visit a doctor… I know few people who go to medical facilities for preventive health care. I don’t go for preventive health care, although I realize that it is necessary. (Female, not screened, Group 4)*

Receiving a personalized invitation can encourage service users to place more value on their own health and remind them that preventative action can be taken to prevent or mitigate disease. A quarter of service users (20/80) mentioned **feeling cared for** upon receiving the invitation. The letter also functioned as a reminder that **screening is an opportunity to prevent complications** and avoid delaying care until urgent attention is required.

*People’s attitude and trust increased a lot due to the intervention, because they had received the documents, and it was something new for them, a different format, a better one. I don’t know, probably people were feeling more appreciated, as someone cared about them and they got an invitation. (Female, Therapist)*

*What can be better than the fact that the state has started to be interested in your health. It makes me happy. (Male, screened, Group 2)*

*Sometimes people have diabetes but don’t know about it, it may be in latent period or may be acquired. If they get such letters, they may attend and avoid further problems. (Female, not screened, Group 2)*

### Theme 2: the decision to attend screening is more likely with greater perceived utility of and access to screenings

Service users who chose to screen often perceive that obtaining information on their disease status was useful, and that subsequent care would provide some health benefit.

Screenings may be perceived as useful if individuals want to know their disease status and seek guidance on how to manage that disease. A quarter (20/80) of service users described **gaining knowledge about their health status** by going to screenings, and sixteen service users also **mentioned seeking medical expertise** about possible conditions. No service users made note of the statistics on neighborhood screening levels that were provided in intervention group 2 and none remembered these statistics when prompted. A proportion of service users (29/80) do not find diagnostic information from screenings helpful, and in fact have a **fear of screening**. To avoid the possibility of knowing that they have a condition, some individuals avoid screenings altogether.

*Screenings are important. There are things you don’t know, and you can’t even imagine, but when you go get screened, something turns out, [proving] you should’ve gone [for screening] a while ago. (Male, screened, Group 3)*

*It was difficult at the beginning; we were explaining people what screenings are for. Now they realize more and even tell each other, ‘You know, I went for screening and found out that I have diabetes, you should also go and be tested.’ Now the attendance is better than at the beginning. (Female, General Practitioner)*

*We always were scared and were avoiding screenings. We cannot accept anything that is new and we were asking why we need this. However, it turns out that we need it. My blood pressure gets high often but I try to ignore it. But when I went for screenings, they told me that I should use medicine. Maybe one day I will be broken as well and will go for screenings on time. (Female, screened, Group 4)*

A screening that yields in diagnosis is only useful if an individual believes that the subsequent care will be beneficial and can be accessed. Thirty service users and five service providers discussed levels of **trust in the health system**, with 22 users expressing doubt towards the expertise of providers or the quality of smaller facilities. Eight service users expressed preference for screening in Yerevan due to increased accuracy or better equipment. Four individuals attributed distrust to past experiences of being **forced to pay bribes** by doctors. Three interviews described **improvements in corruption**, giving credit to the current political administration. The **inability to afford prescribed treatment and continued care** after diagnosis is a major deterrent to deciding to attend screening. Seventeen service users described wanting to attend a preventative screening but finding no purpose in it if they cannot act on new information. Coupled with the psychological burden of being diagnosed, individuals may dismiss the idea of screening altogether. Four service providers supported this sentiment, echoing this mentality among their patients.

*People don’t always believe in testing results and pass them in different places, in Yerevan, in Hrazdan. Test results may be contradictory. People are disappointed because of this; they don’t know who to apply to get correct results. Here in Charentsavan there is a lack of equipment, this also affects testing results, but in Yerevan I think everything is ok. (Female, screened, Group 2)*

*Whenever we visit, they [referring to the staff] are having coffee. Say, a person goes there, opens the door and they go ‘Wait on, we’re drinking coffee.’ What I’m saying is Nikol [referring to Prime Minister Pashinyan] hasn’t visited here, yet. (Male, not screened, Group 1)*

*If I want to go for screenings today, some of the services will cost money and I don’t have it. If I go for screenings and then cannot afford the treatment, why should I go. I prefer to treat myself at home. (Female, not screened, Group 1)*

The interventions of personalized invitation and peer information potentially impacted the perceived utility of screening by **alerting individuals to the information to be gained by attending screenings** and by **increasing trust in the health system**. Ten service users and three service providers commented on the effect of receiving information that peers are attending screenings. They described that learning about others’ screening attendance can make an individual realize that they may be susceptible too, and also remind them that screening is a socially acceptable and positive behavior. Receiving a formal and signed invitation to screening also served to increase trust in the health system, which was often bolstered by a positive experience upon actually attending a screening.

*It is possible that people will think that if others go for screenings, we should also go, that maybe this is the age when a lot of problems occur. And I think that people will go more. (Female, screened, Group 2)*

*I was thinking that this project will help people visit polyclinics and trust the health care system, that they will go for the screenings and will receive the necessary attention, attitude and will be able to solve their health issues. (Female, screened, Group 2)*

### Theme 3: external motivators increase the likelihood of following through with the decision to screen

Receipt of the invitation to screen was itself a **trigger** to consider attending screening, and the formal letter delivered by the fieldworker led to a sense of obligation to follow through with screening. Eleven service users described feeling a sense of **responsibility** after receiving the invitation, and five service providers corroborated this in their observation of patients. Service users felt that they made a promise to fieldworkers or felt the desire to participate in the program they were selected for.

*You should do this more often, which will keep us vigilant and trigger to visit a doctor even without such things. If you take a pause, we forget about it, but when you keep repeating it continually, we start paying more attention. (Female, not screened, Group 2)*

*This is a very good program, because when a nurse calls people, they may not take it serious. But when they get a call from a different organization and they get these letters, this is kind of obligatory for them, they feel more responsible (Female, Director of Polyclinic)*

Pharmacy vouchers were **perceived as motivating** to the service users who received them (26/41) and were viewed by recipients as a **form of financial aid**, ameliorating the cost of care, rather than a signal of screening value, that was modest but useful when needed. Nearly half (41/100) of service users and providers believed the conditional voucher would most likely motivate screening attendance, while less than half of that (15/100) expressed that the unconditional voucher to be adequate in motivating attendance. Service providers noted that the rumor of financial reward for screening was enough to increase screening attendance at the beginning of the program. Further, there were service users in Groups 3 and 4 who received vouchers and attended screenings but expressed they would have attended anyway, even without the voucher. All ten of those in Group 3 who did not screen used their vouchers, or gave them to someone who did, and explained that the voucher was appreciated but they did not feel the need to screen.

*Even without the program I would attend for screenings, and the program was in time, as we purchased necessary things. This also helps people, the program helps giving an opportunity to get some medicine for free, it motivates people to attend screenings. I have not attended just for the money; I would do it anyway. But you should not do it just for the money, it is secondary, it only motivates people more. On the other side, you must have money to purchase medicine for your health. Both are connected to one another. (Female, screened, Group 4)*

*I have received the gift card and did not attend, as I didn’t need it. Those people, who got invitation wouldn’t attend in the same way. The card could not force people to attend for screenings. However, people will be glad to get that card. (Female, screened, Group 3)*

Nine service users commented on the level of need for the vouchers, and how they could be more beneficial to poorer individuals. Further, some service users and one service provider preferred that future iterations of the program expand the vouchers to everyone so that they may be able to afford medicines. In contrast, others were wary of continued assistance and cautioned against creating the expectation of aid.

*I later learnt that some other people from my neighborhood besides the invitation also received vouchers with 5000 AMD. I got angry and I asked myself whether I was not enough poor to receive it as well. (Female, not screened, Group 1)*

*Any pensioner or social assistance beneficiary needed it more than me, but it may form a habit and then they would say: “Why don’t you support me continually?” (Male, not screened, Group 3)*

External motivators such as the invitation and voucher were enough for some service users to overcome the challenge of **finding the time to go** to the clinic and **waiting in queues,** that is the time cost of screening, which was an obstacle for the majority (53/80). Most participants found their local facility easily accessible and acceptable, though the sometimes-preferred larger facilities were often more distant. Service providers in Metsamor also noted that facilities in smaller and more rural areas, might face more challenges in patient attendance.

*Many of them were visiting us for the first time. Once I gathered the staff and we visited people at their homes to make screenings for diabetes, as some people were too busy with their work to attend for screenings. But this time they found time and came. They had a strong belief in you [the program officer]. (Female, Director of Ambulatory)*

## Discussion

This study is one of the few that explores the factors explaining preventive health care use at the primary care level in Armenia. It provides insights that may be relevant for other contexts with low levels of diagnosis of hypertension and diabetes and coincident underutilization of primary care. We also describe the mechanisms through which demand-side interventions, including messages and financial incentives, may be useful in improving the use of preventive health care.

Three themes emerged that appear to explain the decision to attend clinic screenings and follow through on that decision. An individual must first perceive that a screening is needed. This need is informed by the importance placed on their own health, the perception that hypertension and diabetes is sufficiently harmful, and that the conditions can and should be managed. Should an individual choose to take action to prevent and manage hypertension and diabetes, they may seek out facility-based screening if they believe that the encounter will provide useful and wanted information about their disease status and treatment. Screenings may not be perceived as useful if there is a lack of trust in the health system or there are doubts about being able to afford subsequent treatment. External motivators such as a personalized invitation increased the sense of trust in the health system, while the vouchers were viewed as a form of aid that was helpful to purchase medicines and motivating to make time to attend screenings.

While we used the HBM for data collection and analysis, our results expand from some aspects of the HBM in context-specific ways. Novel themes emerged that were specific to explaining care-seeking behavior in Armenia that deviated from the HBM but better synthesized the experience of service users and providers. Our domain of perceived need for screening as a predictor of attendance includes some elements analogous to the HBM’s concept of perceived severity and susceptibility as influencing health behavior. Hence, we find that the perception that hypertension and diabetes is common, potentially harmful, and preventable increases the likelihood of deciding to screen. However, in the Armenian context, we also identified the added dimensions of intrinsically valuing one’s health as predictive of screening behavior. With low priority for one’s health, there is little motivation to act on prevention or treatment even if severity and susceptibility is acknowledged. The concept of perceived utility of screening which increases screening use, is analogous to perceived benefits in the HBM model. In the Armenian context, we highlight that the perceived benefits or utility of screening are reduced by fear of screening and anxiety from being diagnosed with a condition that requires unaffordable treatment. Finally, our domain of following through with the decision to screen identifies the importance of external motivators and reduction in access barriers including time to attend screenings. These concepts are analogous to the cue to action and addressing perceived barriers in the HBM. While we do not aim to build on the HBM, future research on concepts such as the intrinsic valuing of one’s health as a predictor of health utilization can be further investigated with careful field-based research. Exploring whether this concept is specific to Armenia or generalizable to other contexts may be a first step to building on the HBM [47].

Our findings on screening attendance align with other qualitative literature on factors influencing preventive care-seeking behavior in a range of settings. For example, qualitative investigations into care avoidance in the US [48] and preventive care non-attendance in the UK [49] both identified low perceived need for care (or relevance of health checks) as a reason given for not seeking care and found negative past experiences to be a potential contributor to avoidance. Access barriers such as time and financial cost were present in both studies, as factors reducing care use, which is consistent with our findings. In our study, service users reported feeling cared for upon receiving an invitation and that they felt obligated to participate after receiving such attention. Similar results of feeling a responsibility to attend after being asked, or feeling obligated to participate, were found in qualitative explorations of invitations to preventive health checks in Denmark [50] and the UK [51]. Finally, expensive medication was reported as a reason to forgo screenings in our study, since any treatment may be unaffordable following potential diagnosis. In studies on hypertension diagnosis and treatment in Bangladesh [52] and Colombia [53], costs of medication was also identified as a barrier to maintaining treatment, and lapses in treatment were reported as well if symptoms abated. Our study echoed this potential misunderstanding of drug therapy, where medication was reserved for emergent symptoms rather than regulating blood pressure or blood sugar.

Despite the literature on the potential for descriptive social norms to encourage behavior change in applications such as dietary choices [54] or energy-saving behavior [24], there was no qualitative evidence that service users factored the statistics on peer behavior into their own decision to seek care. No service users could recall the specific contents of the invitation letter and spoke more on receiving the invitation itself. However, it is possible that peer screening rates were so low, even in terms of absolute numbers of people who screen, that the information on peer screening habits did not serve as an adequately motivating social norm. In fact, the letter may have even described and reinforced a social norm of low screening rates. Additional research into the impact on descriptive social norms on care utilization should focus on issues with larger differences between the target group and peers.

While service users did not comment on letters containing descriptive social norms, participants from Group 2 and others described learning that neighbors and peers attended screenings as a potential motivator for service users, especially if benefits were evident (such as finding out disease status or getting treatment). A randomized control trial in the UK that tested variations of an invitation letter on screening attendance, including one with descriptive social norms, found similar results where the letter with descriptive norms did not perform better than other versions in increasing attendance [21]. In the context of our study, absolute numbers were used to describe peer screening rather than percentages, since highlighting the low percentages of attendance was not considered to be motivating information. If replicated, further research should explore potential differences in forms of social norm messaging. In addition, peers were defined in our study to refer to neighbors within a similar age range. It may be, however, that these are not the comparators that are relevant to the average Armenian. Future research on this subject should include formative research to characterize the profile of peers, information on whom may influence screening behavior.

Conditional and unconditional cash transfers, or its variants such as vouchers, have been tested in multiple demand-side interventions aiming to incentivize health care behaviors [55]. They have been shown to increase levels of the desired behavior, with conditional cash transfers yielding greater initial impacts than unconditional cash transfers [56]^.^ Perceptions of service users and providers were in line with this sentiment, where participants believed that Group 4 (conditional vouchers) would be more motivated to attend screenings to receive the pharmacy voucher. Participants described that despite the modest amount, the voucher was meaningful and relieved them of significant medication costs. This responsiveness to vouchers confirms that medication costs are a burden for many Armenian families, and increasingly so for lower income families [57]^.^ However, it should also be noted that while those in Groups 3 and 4 expressed that vouchers motivate attendance, some in Group 3 used the vouchers but did not screen because they did not feel the need. In contrast, some participants of the both voucher groups attended screenings and claimed they would have done so regardless of the voucher. While participants may state this is the case, quantitative data showed higher attendance under Group 4 and sheds light on the value of conditionality in increasing targeted health behaviors [38].

Providers observed an increase in screening attendance by those who merely heard of the program and promise of financial reward. This observation indicates demand for the voucher program beyond this trial but also raises questions of ethics for intervention scale-up, such as equity in access to vouchers that improve affordability of necessary medications and supplies. Both service users and providers commented on the greater need for such vouchers among poorer groups, and some proposed that everyone be eligible for vouchers if the program were expanded. Any plans to scale up the program should consider the acceptability and ethics of using financial incentives to influence health-related decision making, in addition to the non-financial nudges of invitations and social norms messaging. Further research is needed to determine the acceptability of such nudges for health programs in Armenia, in addition to other criteria such as sustainability and cost, and address any concerns on manipulation of decision-making that has been raised in debates on the ethics of nudges [58, 59]. As acceptability of such programs varies by country [60, 61], program modifications can be made for the Armenian context to ensure that choices and accompanying consequences are sufficiently transparent and fair to potential service users in Armenia.

Our qualitative investigation suggests that personalized invitations from physicians are simple but effective policy measures to encourage screening attendance by prompting individuals to reassess their need for screening and consider their local facility as an acceptable option to do so. Including information on peer screening rates, if they are not sufficiently different from the individual’s screening behavior, may not enhance the effect of an invitation perceptibly. An additional pharmacy voucher will likely be appreciated and used by recipients, but attendance may not appreciably increase relative to messages if the voucher is not conditional on it. Hence, from a policy perspective, personalized invitations are a potentially sustainable and acceptable intervention to increase screening attendance. While vouchers are effective, they require resources to sustain. In the cost-effectiveness analysis of intervention types, the conditional vouchers were equally cost-effective as messaging interventions, while the unconditional vouchers were less cost-effective than the other interventions [38]. Feedback from participants highlights the importance of addressing high out-of-pocket costs of hypertension and diabetes treatment that follows a diagnosis, including outpatient medicines. The costs are prohibitive enough to discourage screening even if there is perceived need, since treatment would be unobtainable in the event of diagnosis.

Our study also highlights the importance of policies to expand education on hypertension and diabetes risk and management. As evidenced by sporadic treatment with medication or home remedies, individuals may not fully understand hypertension or diabetes as conditions where blood pressure or blood sugar levels should be maintained rather than treated emergently. Given the registration of 85 to 100% of the population with a primary care provider, and the increase in screening attendance following messages from a family physician, these may be effective channels for tailored messages to target groups on the risks and management of NCDs. Our study highlights the importance of personalized messages in promoting preventive care use which had additional value in a context where mass media campaigns had been conducted for many years.

Our study has some limitations. Because our sample was restricted to participants of the randomized control trial that selected individuals who had not screened in the previous 12 months, our results may not be generalizable to individuals who differ in terms of screening behavior. Another limitation is our exclusion of control group participants, which may have illustrated any differences in care-seeking preferences in the absence of this intervention program. It is also difficult to evaluate any impact of the Group 2 invitation where descriptive social norms were used, as the peer screening rates may have been too low to motivate participants. Future research should further explore the element of facility size and its perceived acceptability. Individuals may believe that seeking care is more burdensome if they overlook their local facility and have greater trust in a larger but more distant (often urban) facility. Trust in the health system should also be studied, especially in relation to health seeking behaviors and recent reforms to reduce corruption.

## Conclusion

This qualitative study explores the mechanisms through which various demand-side interventions can increase attendance at facility-based hypertension and diabetes screenings in Armenia. Deciding to seek health care services such as screening depends on an individual’s perceived need to screen, the perceived utility of screening, and any external motivators that impact following up on the decision to screen. Personalized invitations can encourage screening attendance because they alert individuals to the potential need to prevent or manage hypertension and diabetes. Vouchers are appreciated by individuals who often have considerable medication costs and incentivizes attendance if voucher receipt is conditional on it. Invitations and conditional vouchers are recommended policy options to increase screening attendance, which can be augmented with other policies targeting health education and out-of-pocket medication expenditure.

## Supplementary Information

**Additional file 1.**

**Additional file 2.**

## Data Availability

The datasets generated and analyzed during the current study are not publicly available due potentially identifiable information but are available from the corresponding author on reasonable request.

## Abbreviations

HBM: Health Belief Model
LMIC: Lower- and middle-income country
NCD: Non-communicable disease
