# Supplementary material for: Invitations and incentives: a qualitative study of behavioral nudges for primary care screenings in Armenia
Source: BMC Health Serv Res. 2020 Dec 1;20:1110. doi: 10.1186/s12913-020-05967-z (PMC7709231; doi:10.1186/s12913-020-05967-z)
Supplement: Supplementary file 1 — Additional file 1. [file 12913_2020_5967_MOESM1_ESM.docx]

**Personal nvitation (Intervention group 1)**

Dear [Name, Surname],

We cordially invite you to visit [Name of the medical facility] of [Name of the community] at the following address [Address] to be screened for diabetes and hypertension.

According to official records in your personal medical card, in past 12 months you have not been screened for diabetes and hypertension at a medical facility. In terms of prevention and control of diseases, your visit to the medical facility is extremely important to ensure your personal healthcare.

Please, prior to the visit make a call to your doctor for a proper appointment, using the telephone number presented in the bottom of this invitation.

***Note:*** for a credible measurement of diabetes you must visit the doctor fasting since midnight, which means you should not eat or drink anything except water. The screening is free-of-charge, painless and not time-consuming.

We highly recommend that you visit the medical facility soonest possible and to use the free-of-charge medical services of the primary healthcare facility.

Looking forward to seeing you,

| DATE |  |
| --- | --- |
| DOCTOR |  |
| SIGNATURE |  |

**Personal invitation with peer group information (Intervention group 2)**

**X**

**Y**

Dear [Name, Surname],

We cordially invite you to visit [Name of the medical facility] of [Name of the community] at the following address [Address] to be screened for diabetes and hypertension.

According to official records in your personal medical card, in past 12 months you have not been screened for diabetes and hypertension at a medical facility. In terms of prevention and control of diseases, your visit to the medical facility is extremely important to ensure your personal healthcare.

**We would like to inform you that based on official statistics for your community among your peers, X men and Y women have already taken this screening, and what about you?**


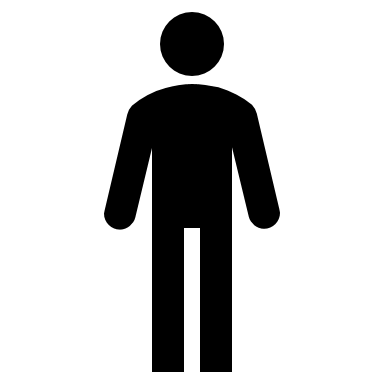

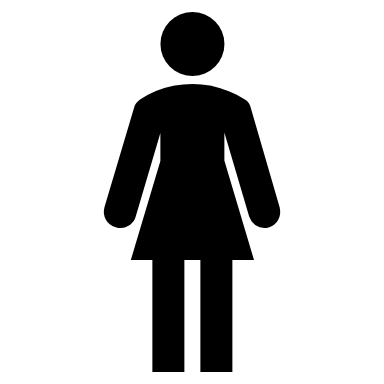


Please, prior to the visit make a call to your doctor for a proper appointment, using the telephone number presented in the bottom of this invitation.

***Note:*** for a credible measurement of diabetes you must visit the doctor fasting since midnight, which means you should not eat or drink anything except water. The screening is free-of-charge, painless and not time-consuming.

We highly recommend that you visit the medical facility soonest possible and to use the free-of-charge medical services of the primary healthcare facility.

Looking forward to seeing you,

| DATE |  |
| --- | --- |
| DOCTOR |  |
| SIGNATURE |  |

**Personal invitation with a labelled pharmacy voucher (Intervention group 3)**

Dear [Name, Surname],

We cordially invite you to visit [Name of the medical facility] of [Name of the community] at the following address [Address] to be screened for diabetes and hypertension.

According to official records in your personal medical card, in past 12 months you have not been screened for diabetes and hypertension at a medical facility. In terms of prevention and control of diseases, your visit to the medical facility is extremely important to ensure your personal healthcare.

**To encourage your participation in screening, you will receive a single-usage voucher, which allows you to purchase free-of-charge medication or medical products for an amount equivalent to 5000 AMD. The voucher can be used at the below-mentioned pharmacies:**

| Natali Pharm Chain | Alfa Pharm Chain |
| --- | --- |
| Address | **Address** |
| Address | **Address** |

**In case of questions related to the utilization of the voucher, you can call the number mentioned on it. There is a unique ID on the voucher for verification. Again, if you present the voucher at any of above-mentioned pharmacies you can receive for free medicine or medical products that you prefer. The voucher is valid till [Date]. If lost, the voucher cannot be recovered.**

**We very much hope that the voucher will encourage you to visit our medical facility as soon as possible**. Please, prior to the visit make a call to your doctor for a proper appointment, using the telephone number presented in the bottom of this invitation.

***Note:*** for a credible measurement of diabetes you must visit the doctor fasting since midnight, which means you should not eat or drink anything except water. The screening is free-of-charge, painless and not time-consuming.

We highly recommend that you visit the medical facility soonest possible and to use the free-of-charge medical services of the primary healthcare facility.

Looking forward to seeing you,

| DATE |  |
| --- | --- |
| DOCTOR |  |
| SIGNATURE |  |

**Personal invitation with a conditional pharmacy voucher (Intervention group 4)**

Dear [Name, Surname],

We cordially invite you to visit [Name of the medical facility] of [Name of the community] at the following address [Address] to be screened for diabetes and hypertension.

According to official records in your personal medical card, in past 12 months you have not been screened for diabetes and hypertension at a medical facility. In terms of prevention and control of diseases, your visit to the medical facility is extremely important to ensure your personal healthcare.

**To encourage your participation in the screening, within one week after your visit to the medical facility and being screened for diabetes and hypertension by the medical staff, you will receive a single-use voucher, which allows you to receive free-of-charge medication or medical products for an amount equivalent to 5000 AMD. Any questions related to the utilization of the voucher will be explained to you individually.**

**We very much hope that the opportunity to receive free-of-charge medicine or medical products will encourage you to visit our medical facility soonest possible.** Please, prior to the visit make a call to your doctor for a proper appointment, using the telephone number presented in the bottom of this invitation.

***Note:*** for a credible measurement of diabetes you must visit the doctor fasting since midnight, which means you should not eat or drink anything except water. The screening is free-of-charge, painless and not time-consuming.

We highly recommend that you visit the medical facility soonest possible and to use the free-of-charge medical services of the primary healthcare facility.

Looking forward to seeing you,

| DATE |  |
| --- | --- |
| DOCTOR |  |
| SIGNATURE |  |
