# Supplementary material for: Invitations and incentives: a qualitative study of behavioral nudges for primary care screenings in Armenia
Source: BMC Health Serv Res. 2020 Dec 1;20:1110. doi: 10.1186/s12913-020-05967-z (PMC7709231; doi:10.1186/s12913-020-05967-z)
Supplement: Supplementary file 2 — Additional file 2. [file 12913_2020_5967_MOESM2_ESM.docx]

**Qualitative interview guide for service users**

INTERVIEWEE CHARACTERISTICS

| **Province code** | **Medical Facility Rank**   1. **small** 2. **medium** 3. **large** | **Medical Facility/ Settlement Code** | **Cluster**   1. **Intervention group 1** 2. **Intervention group 2** 3. **Intervention group 3** 4. **Intervention group 4** | **ID** | **Gender** | **Age** |
| --- | --- | --- | --- | --- | --- | --- |
|  |  |  |  |  |  |  |

INTERVIEWER’S CHARACTERISTICS

| **Name** | **Gender** | **Age** | **Background education** | **Academic record** |
| --- | --- | --- | --- | --- |
|  |  |  |  |  |

INTERVIEW SETTING

| **Date [day. month]** | **Interview duration [hh:mm]** | **Place of interview, privacy, etc.** |
| --- | --- | --- |
|  |  |  |

INTERVIEWER: MAKE SURE THAT THE INTRODUCTORY TEXT IS READ OUT AND THE CONSENT FORM IS SIGNED BY THE RESPONDENT.

Good day, my name is___________________ (Name, Surname): I’m representing Media-Model LLC, which is implementing an impact evaluation for the World Bank project “Comparing different demand-side incentives for health screenings in Armenia”. In the summer of 2019, we contacted you asking to participate in the project. Our field officers visited you to familiarize with the project and you expressed your willingness to participate, signing a consent form. The project is now on its final stage, and we are re-visiting some of our participants to collect feed-back and insights. I would like to discuss with you in more detail some aspects of the project, which are very important for this impact evaluation. Your participation today is voluntary and confidential. You may reject to answer any questions or to continue the interview any time you wish. I hope that you will feel free to express your opinions and share your experience with me. There are no right or wrong answers. Please, feel free to give long and detailed answers. With your permission, the interview will be audio-recorded for accurate and full transcription of the information. My assistant will be making some notes. This information will be used for analytical purposes only, and all identifiable information such as names or contact information will be removed. Once the study is completed data from the study will be generalized and presented as summarized analysis. Your participation is extremely important for future improvement of programs on healthcare for people in your community and in Armenia. The interview will take about 30-40 minutes.

Do you have any questions before we start? May I turn on the audio-recorder?

**SECTION ONE: PERCEPTIONS OF HYPERTENSION AND DIABETES TESTING BEFORE THE INTERVENTIONS**

1. Could you please recall the period before our staff visited you in summer of 2019 and tell me what did you know about hypertension and diabetes testing at that time?

***INTERVIEWER: Probe asking about:***

- Sources of information
- Stories heard
- Experiences within the family and around in the community
- Importance of testing
- Where and how to take screening

1. In your opinion, how often usually people in your community go for health screenings? Why? What is making some people to go for testing more often than the others? What is the difference in case of curative and preventive healthcare?

***INTERVIEWER: Probe asking about:***

- - People of different age groups (youth, middle age, older people)
  - People of different gender (men versus women)
  - People of different status, education, other

Did the intervention change this perception? How?

1. Who are main groups or people in your community that can influence your and other people’s behavior, making you to go for medical screening more often? (***INTERVIEWER: Probe asking about: family, peers, local authorities, media***) What should they do or say to change the behavior?
2. Based on our records, prior to the visit of our field officers to your HH, you had not taken medical screening for diabetes and/or for hypertension during the previous 12 months at this particular medical facility (**INTERVIEWER: READ OUT THE NAME OF THE FACILITY)** Could you please tell me why?

***INTERVIEWER: Probe asking about:***

- Main reasons
- Fears or stereotypes
- Perceived costs for screening
- Perception of service readiness and quality
- Physical access to facility, (including hours of operation, waiting times, etc.)
- Discrimination/attitude of the staff (does the service delivery interaction vary in unacceptable ways with service user characteristics?).
- Other circumstances

**SECTION TWO: PERCEPTIONS OF THE INTERVENTION**

1. Could you please recall the day when our staff visited you for the first time in summer 2019? What did you like about the visit? What did you dislike about the visit? What kind of feelings/thoughts about the project did you have? ***INTERVIEWER: AKS FOR EXAMPLES.***
2. Do you have any suggestions to changing/improving the intervention? ***INTERVIEWER: Probe asking about:***

- Were the documents clear enough? What could be improved?
- Were the officers’ attitude and instructions clear enough? What could be changed in this regard?
- What do you think about the four groups of interventions and the way you were assigned to this particular Group **(INTERVIEWER: READ OUT THE NAME OF THE GROUP)?**
- What was your impression after you read the invitation for your particular group? **INTERVIEWER: DEPENDING ON THE GROUP PROBE MORE**
  - GROUP ONE: opinion about the personal invitation and reaction
  - GROUP TWO: opinion about the personal invitation with statistics and reaction, understanding of statistics **INTERVIEWER: USE PROMPTS ASKING:** Did the statistics differ from your initial perception of how often people in your community go for screenings? Did the statistics change your perception of how often people in your community go for screenings? How? Does peer behavior matter for your testing? How? Is there a difference for some health care and not other, e.g. preventive versus curative care?
  - GROUP THREE: opinion about the personal invitation and voucher gift, reactions, **INTERVIEWER: USE PROMPTS ASKING:** Did specifying that the voucher with the money was given to encourage going for screening, change the way you perceived it? In what way?
  - GROUP FOUR: opinion about the personal invitation and conventional voucher, reactions **INTERVIEWER: USE PROMPTS ASKING:** Did specifying that the voucher with the money was given to encourage going for screening, change the way you perceived it? In what way?

1. **IF FROM GROUPS THREE OR FOUR ASK:** How did you find the voucher system: was it convenient and efficient? How did you use your voucher? What kind of products did you purchase? Why? What you liked about it? What you didn’t like about it? How the voucher influenced your desire to take screening? Did it change perception of the service provided by medical facility? If yes, in what way? Why?
2. In your opinion which group will have better screening performance? Why do you think so?

**SECTION THREE: PERCEPTIONS OF HYPERTENSION AND DIABETES TESTING AFTER THE INTERVENTIONS**

1. Could you please tell me what happened after you received the invitation /IF FROM GROUPS THREE OR FOUR: and the voucher?

***INTERVIEWER: Probe asking:***

- - Did it change your perception of the importance to go for screening in comparison to the period before we contacted you? Why? How?
  - Did it change your knowledge about the screenings:
    1. where and how to take screening,
    2. costs for screening,
    3. fears or stereotypes
  - Did you go for testing because of the invitation /IF FROM GROUPS THREE OR FOUR: and the voucher? If yes, why? If not, why not?
  - Did you discuss it with your family, other people around? What kind of discussions took place?

1. **IF WENT FOR SCREENING:** Please, tell me about your experience at the facility where you took the screening for the diabetes and hypertension?

***INTERVIEWER: Probe asking about:***

- - Timing and duration
  - Attitude of doctors and other medical staff
  - Infrastructure and equipment
  - Peculiarities/differences in terms of:
    1. Age groups,
    2. Gender,
    3. economic status, etc.
  - Other circumstances

1. What should be changed/improved in the way the screenings for hypertension and diabetes are offered to people in your community?
2. What are your plans for screening of hypertension and diabetes 12 months after your last screening? Why?
3. Do you want to add anything that we didn’t discuss?

**THANK YOU**

**Qualitative interview guide for service providers**

INTERVIEWEE CHARACTERISTICS

| **Province code** | **Medical Facility Rank**   1. **small** 2. **medium** 3. **large** | **Medical Facility/ Settlement Code** | **Gender** | **Age** | **Position** | **Years in service** |
| --- | --- | --- | --- | --- | --- | --- |
|  |  |  |  |  |  |  |

INTERVIEWER’S CHARACTERISTICS

| **Name** | **Gender** | **Age** | **Background education** | **Academic record** |
| --- | --- | --- | --- | --- |
|  |  |  |  |  |

INTERVIEW SETTING

| **Date [day. month]** | **Interview duration [hh:mm]** | **Place of interview, privacy, etc.** |
| --- | --- | --- |
|  |  |  |

INTERVIEWER: MAKE SURE THAT THE INTRODUCTORY TEXT IS READ OUT AND THE CONSENT FORM IS SIGNED BY THE RESPONDENT.

Good day, my name is___________________ (Name, Surname): I’m representing Media-Model LLC, which is implementing an impact evaluation for the World Bank project “Comparing different demand-side incentives for health screenings in Armenia”. In summer 2019 we have contacted your facility asking to participate in the project. Our field officers visited you to familiarize with the project and you supported the team to sign the invitations to the population to visit your facility for screenings. The project is now on its final stage, and we are re-visiting some of the facilities to collect feed-back and insights. I would like to discuss with you in more detail some aspects of the project, which are very important for this impact evaluation. Your participation today is voluntary and confidential. You may reject to answer any questions or to continue the interview any time you wish. I hope that you will feel free to express your opinions and share your experience with me. Please, feel free to give long and detailed answers. With your permission, the interview will be audio-recorded for accurate and full transcription of the information. My assistant will be making some notes. This information will be used for analytical purposes only, and all identifiable information such as names or contact information will be removed. Once the study is completed data from the study will be generalized and presented as summarized analysis. Your participation is extremely important for future improvement of programs on healthcare for people in your community and in Armenia. The interview will take about 30-40 minutes.

Do you have any questions before we start? May I turn on the audio-recorder?

**SECTION ONE: PERCEPTIONS OF THE INTERVENTION**

1. Our team asked for your support to sign the invitations and to provide up-screening progress reports. What kind of experience was it? What kind of difficulties did you face?
2. What is your general opinion about the project and the interventions proposed for the patients? What did you like about it? What did you dislike about it? Why? ***INTERVIEWER: AKS FOR EXAMPLES.***
3. Have you heard about any other interventions (outside ours) aimed at increasing medical screening of the population in your community? And in the country? (INTRVIEWER: Ask about interventions from the state, on the facility-level, other non-state bodies). Could you please tell what have or have not worked in boosting screening? Why it happened?
4. Do you have any suggestions to changing/improving this particular intervention? ***INTERVIEWER: Probe asking:***

- Were the documents clear enough? What could be improved?
- Were the officers’ attitudes and instructions good enough? What could be changed in this regard?

1. What do you think about the four groups of intervention? And the way patients were assigned to a particular Group? What kind of feed-back did you receive from the patients? ***INTERVIEWER: AKS FOR EXAMPLES RE GROUPS ONE, TWO, THREE AND FOUR.*** Did it change perception of the service provided by medical facility? If yes, in what way? Why?
2. In your opinion which group will have better screening performance? Why do you think so?
3. How did you find the voucher system? How did you use your voucher? What kind of products did you purchase? Why? What you liked about it? What you didn’t like about it?

**SECTION TWO: PERCEPTIONS OF HYPERTENSION AND DIABETES TESTING AFTER THE INTERVENTIONS**

1. In your opinion, how often usually people in your community go for health screenings? Why? What is making some people to go for testing more often than the others? What is the difference in case of curative and preventive healthcare?

***INTERVIEWER: Probe asking about:***

- - People of different age groups (youth, middle age, older people)
  - People of different gender (men versus women)
  - People of different status, education, other

1. Could you please tell me how the intervention changed the perceptions of people for testing?
   - Did it change their perception of the importance to go for screening in comparison to the period before we contacted them? Why? How?
   - Did it change the knowledge about the screenings? ***INTERVIEWER: Probe asking about:***
     1. where and how to take screening,
     2. costs for screening,
     3. fears or stereotypes
   - Did patients go for testing because of the personal invitation? Because of the vouchers? If yes, why? If not, why not?
2. Who are main groups or people in your community that can influence people’s behavior, making them to go for medical screening more often? (***INTERVIEWER: Probe asking about: family, peers, local authorities, media***) What should they do or say to change the behavior?
3. What should be changed/improved in the way the screenings for hypertension and diabetes are offered to people in your community?

***INTERVIEWER: Probe asking about:***

- - Timing and duration
  - Attitude of doctors and other medical staff
  - Infrastructure and equipment
  - Peculiarities/differences in terms of:
    1. Age groups,
    2. Gender,
    3. economic status, etc.
  - Other circumstances

1. Do you want to add anything that we didn’t discuss?

**THANK YOU**
